# The characteristic analysis of phase-controlled array thermo-acoustic emission with multiple emitting surfaces

**DOI:** 10.1038/s41598-023-48168-4

**Published:** 2023-11-24

**Authors:** Kai Zhang, Dongdong Wang, Jiayi Zhou, Yulei Su, Huaikuang Ding, Hanping Hu, Yuanzhao Han

**Affiliations:** 1 Vacree Technologies Co., Ltd., Hefei, 230088 China; 2 Department of Thermal Science and Energy Engineering, University of Science and Technology of China, Hefei, 230027 Anhui China

**Keywords:** Energy science and technology, Nanoscience and technology, Applied physics

## Abstract

Thermo-acoustic (TA) ultrasound, particularly when combined with phased-controlled array technology, has garnered significant interest in the past decade due to its numerous advantages. This paper establishes a theoretical expression for thermo-acoustic phased array (TAPA) emission to investigate different acoustic fields based on input heat flow frequencies, quantities and distances of TA emission surfaces, area of emission surfaces, and phase changes between emission surfaces. The study finds that a TAPA with two emitting surfaces in a line pattern produces a consistent acoustic field compared to a single emitting surface arranged in a semicircle. Additionally, applying different phases on the surfaces narrows the scanning range with an increase in frequency, area of the TA emission surface, and the amount of emission surfaces, while enhancing the directivity of the TA wave. Moreover, increasing the distance between emitting surfaces in a square-shaped TAPA does not affect the ultrasound pressure of the main TA ultrasound but increases the quantity and size of side lobes. Furthermore, enlarging the area of emitting surfaces enhances the directivity of the TA ultrasound, necessitating a reduction in the distance between emitting surfaces or an increase in the area of the emitting surfaces in a square-shaped TAPA to enhance directivity. This paper provides a comprehensive study of TAPA to aid further research in this field.

## Introduction

In 1879, W. H. Preece made the first observation of the sound produced by a metal wire or thin metal sheet due to the thermal effect of electric current and conducted experimental comparisons of the sound magnitude produced by different conductive wires or metal sheets^1^. Subsequently, Arnold and Crandall experimentally investigated this thermo-acoustic (TA) phenomenon by introducing an alternating current into a suspended 700-nm-thick thin platinum metal film. They established theoretical formulas and physical models to explain the phenomenon^2^. Later, Wente^3^ suggested that this TA phenomenon could be utilized to measure the thermal conductivity of gases, while other scholars sought to study the limits of human hearing based on the TA phenomenon^4^.

The TA ultrasound are attractive for use in acoustical device for its rich spectral features in the wide range of amplitude-frequency response, which first proposed to put a conducting 30 nm-thin aluminum film on porous silicon (PS) as TA ultrasonic source by Shinoda et al. in 1999^5^. Subsequent research has delved into various aspects of TA ultrasound. Migita et al.^6^ found that TA signals exhibit stability in long-time continuous wave operation and a fast response capability in a relatively wide frequency range not only for pulsed but also impulsed operation in 2002. Asamura et al.^7^ found that the TA pressure generated by thermally induced ultrasonic emissions can be used on non-contact actuators at the same time. And Tsubak et al.^8,9^ studied the relationship between the porosity of porous silicon and the size of acoustic ultrasound emitted by TA ultrasound in 2005.

Moreover, Various theoretical advancements has been put forward. For example, Xiao et al.^10^ improved on Arnold and Crandall’s model by considering the effect of heat capacity per unit area for the first time. Shinoda and Koshida et al. used McDonald and Wetsel's thermo-elastic coupling equation for photo acoustic effects to derive an expression for the acoustic pressure of TA emission from porous silicon, but the theory does not take into account the thermal coupling in gases^11,12^. Our team also conducted theoretical research around the TA emission, deriving a series of TA equations using a fully thermally-mechanically coupled TA model^13–16^. Based on the above-mentioned theory, Wang et al.^17^ studied the characteristics and regularities of ultrasound from spherical focusing TA emitter in detail. Hirota et al.^18^ investigated the emission characteristics of TA emission from one-dimensional porous silicon arrays and showed that the directivity of TA waves can be achieved by changing the frequency of the input current. Gelloz et al.^19^ arranged nine porous silicon TA emission surfaces into a 3 × 3 array to study the effect of changes in emission frequency and phase on the directivity of its TA emission, and concluded that proper adjustment of the size of the array element, the density of the array, as well as the relationship between the input frequency and the phase can be realised by concentrating and adjusting the direction of the emission density. Watabe et al.^20,21^ investigated the changes in the characteristics of TA waves emitted by thermally induced ultrasound of porous silicon under pulse driving and found that the directivity of the TA pressure increases with the decrease of the pulse width, whereas the amplitude of the TA pressure is independent of the width of the pulse. Tsubaki et al.^22^ found that the images obtained by thermal ultrasound detection have higher spatial resolution compared to conventional piezoelectric ultrasound. Zhou et al.^23^ concludes that the CNT thin film spherical array panel can generate focused ultrasound beam with adjustable focal region, satisfactory ultrasound intensity.

However, existing research on TAPA is still incomplete, particularly regarding the effect of heat flow frequencies, number, distance, and phase changes of TA emitting surfaces. This work aims to study thermally induced ultrasonic emission from phased arrays using a thermally-mechanically coupled TA model. By deriving a comprehensive solution for the acoustic field, systematic investigations on TAPA can be conducted, offering significant insights into understanding TAPA features and guiding further experimental searches.

## Methods

### Theoretical formulas and solution

The derived equation of the TA emission from a surface source consists of the backing, ample and heating film, and considering the element surface $\Omega_{i}$, phase difference $\theta_{i}$ and the gas heat distribution factor, (*i* = 1, 2, 3…)^14,24^:

1 $$ p_{g} = \sum\limits_{i} {\iint\limits_{{\Omega_{i} }} {\frac{j\omega }{{2\pi r}}\frac{\gamma - 1}{{v_{g}^{2} }}}} \frac{{e_{g} }}{{Me_{s} + e_{g} + \sqrt {j\omega } C_{f} }}q_{0} e^{{ - j\left( {\frac{\omega r}{{v_{g} }} + \theta_{i} } \right) - \frac{{\omega^{2} (\gamma - 1)\alpha_{g} r}}{{2v_{g}^{3} }} - \frac{{2\omega^{2} \mu_{g} r}}{{3\nu_{g}^{3} \rho_{g} }}}} d\Omega_{i} $$

where $$M = \frac{{e^{{\sigma_{s} L_{s} }} (\kappa_{b} \sigma_{b} + \kappa_{s} \sigma_{s} ) + e^{{ - \sigma_{s} L_{s} }} (\kappa_{b} \sigma_{b} - \kappa_{s} \sigma_{s} )}}{{e^{{\sigma_{s} L_{s} }} (\kappa_{b} \sigma_{b} + \kappa_{s} \sigma_{s} ) - e^{{ - \sigma_{s} L_{s} }} (\kappa_{b} \sigma_{b} - \kappa_{s} \sigma_{s} )}}$$

$$ \sigma_{b} = \sqrt {\frac{j\omega }{{\alpha_{b} }}} $$

$$ \sigma_{s} = \sqrt {{{j\omega } \mathord{\left/ {\vphantom {{j\omega } {\alpha_{s} }}} \right. \kern-0pt} {\alpha_{s} }}} $$

subscript *b, s* stand for backing and sample respectively.* L*_*s*_ is the thickness of the sample, and *r* is the distance from TA source to the measure point. $v_{g} = \sqrt {{{\gamma P_{0} } \mathord{\left/ {\vphantom {{\gamma P_{0} } {\rho_{g} }}} \right. \kern-0pt} {\rho_{g} }}}$ is the sound velocity in gas [m/s], *γ* is the specific heat ratio of gas, *κ*_*i*_ is the thermal conductivity [W m^−1^ K^−1^], and thermal diffusivity *α*_*i*_ = *κ*_*i*_/*ρ*_*i*_*c*_*p,i*_ [m^2^ s^−1^], density *ρ*_*i*_ [kg m^−3^], specific heat *c*_*p,i*_ [J/(kg K)], thermal effusivity $e_{i} = \sqrt {k_{i} \rho_{i} c_{p,i} }$, subscript i can take s, g, and f for the sample, gas, and heating film, respectively.$C_{f} = \rho_{f} c_{f} \delta_{f}$, *δ*_*f*_ is the thickness of the heating film, $q_{0}$ is the amplification [W/m^2^].

**Figure 1:**
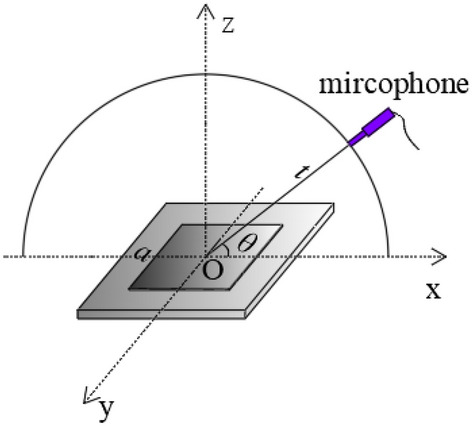
Schematic of one TA emission surface, *t* is the distance from the measuring position to the midpoint of the TA emission surface line,θ is the altitude angle, a is the length of the square emission surface.

In the following analysis of the characteristic of the TAPA from the single TA emission surface, the line pattern TA type and the square shape TA type, we assumed that the nanothermophone is placed in the air at normal atmospheric pressure and room temperature. Its schematic is shown in Fig. 1.

## Results and discussions

### The TA emission of a single emitting surface

The comparison between theoretical and experiments results has been verified by Hu^24,25^. Thus, the related studies based on Eq. (1) can be directly utilized, as shown in Fig. 1. In our study, we used a three-layer structure with silicon as the substrate, porous silicon as the sample, and coated with a heated aluminum film. The relevant parameters are listed in Table 1.

**Table 1:** Relevant physical parameters^26–29^.

Materials	Silicon (Si)	Porous silicon (Ps)	Air	Aluminium film (Al)
Dencity *ρ* (kg m^−3^)	2.33 × 10^3^	0.699 × 10^3^	1.16	2702
The thermal conductivity *κ*_*g*_ × 10^2^ (W m^−1^ K^−1^)	16,800	100	2.63	23,700
Thermal diffusion coefficient *α*_*g*_ × 10^6^ (m^2^ s^−1^)	100	1.43	22.5	97.1

Using the formulas, we studied the acoustic properties of a single TA emission surface, analyzing the ratio of the lengths of different TA emission surfaces and the wavelength of the input frequency. The applied heat flow is *q*_0_ = 25W/cm^2^, thus, the TA signals on a semicircular circumference *t* = 60 mm from the centre of the emitting surface is displayed.

**Figure 2:**
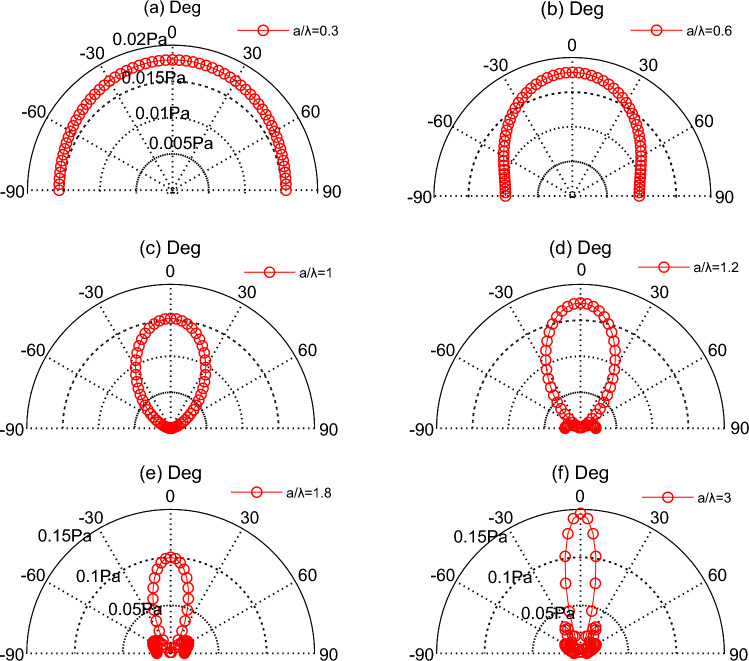
TA sound pressure directivity at different *a/λ* at the area of 2 mm × 2 mm.

**Figure 3:**
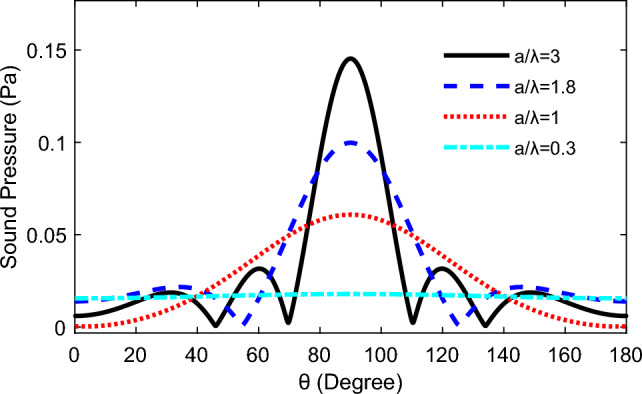
TA sound pressure change with angle.

The directivity of the TA sound is shown in the Fig. 2a respectively for a single square TA emitter with the slide length 2 mm, and the input frequency is approximately 50 kHz. In this case, the TA signal is uniformly distributed on a semicircle 60 mm from the centre of the TA emission surface, regarded as a point source. As the ratio of *a/λ* increases, the distribution of the TA signal in the 60 mm semicircle becomes similar to an ellipsoid under the input frequency *f* = 174 kHz and the *a*/*λ* = 1, which displayed in Fig. 2c. At the same time, the directivity has been revealed, and there is no interference from the side lobes. As the ratio of *a*/*λ* increased, the side lobes begin to appear, but the directivity of the TA emission of the square emitting surface is also becoming progressively evident. Additionally, the sound pressure is concentrated above the emitters, and the side lobes increased with the enlargement of the ratio *a*/*λ.* Moreover, the regularity in the above analysis also can clearly described in the Fig. 3.

**Figure 4:**
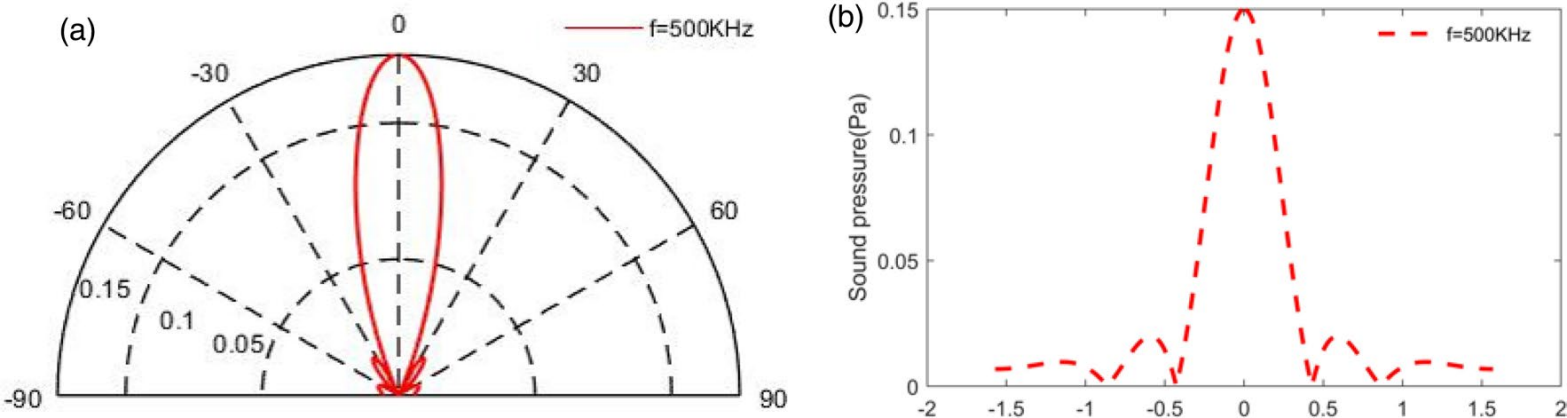
TA sound pressure directivity and sound pressure based on the RB formula in the Ref^30^: (**a**) the directivity at 500KH; (**b**) sound pressure at 500 kHz when *a*/*λ* is 3.

The commonly-used RB model for sound directivity was compared with TA model in this section. The directivity in Fig. 2f is same to the Fig. 4a and similarly, the results in Fig. 3 is consistent with the survey in Fig. 4b. We compared these two models, and as expected, our model produces an acoustic field that matches what would be predicted by a theoretical circular piston in a rigid planar baffle, which illustrates the accuracy of the present model from the vision of acoustic field, including the directivity and side-lobs based on the single emitting surface.

### The research of acoustic field of TAPA

**Figure 5:**
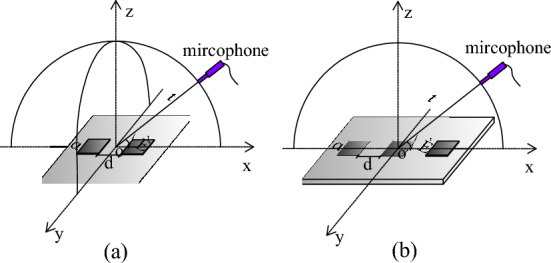
Basic schematic of TA emission surfaces arranged in odd or even with one-line pattern.

In the previous section, we studied the behavior of a single TA emitter. However, TAPA is realized by multiple surfaces. The arrangement of an even or odd quantity of TA emitting surfaces in a line is represented by two or three emitters, as shown in Fig. 5, with the distance between the emitting surfaces denoted as *d* and the side length of the unit TA emitting surfaces as *a*. The two emitting surfaces and three TA emitting surfaces array are investigated in detail, respectively.

#### The sound pressure distribution of the two emitting surfaces

As showed in Fig. 5a, its a simple array of two TA emission surfaces, the TA signal to the origin of the coordinates is *t,* and because the array is one-dimensional, the different TA sound field distribution would occur in yoz plane and the xoz plane.

**Figure 6:**
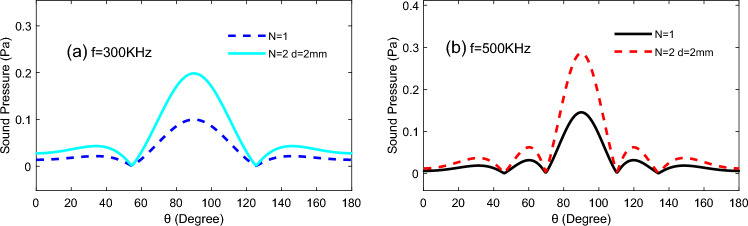
Curves of TA sound pressure in one and two TA emitter change with angle of yoz plane: (**a**)* d* = 2 mm, *f* = 300 kHz; (**b**) *d* = 2 mm, *f* = 500 kHz.

We observed the distinction of the model in Fig. 5a, and the result is displayed in Fig. 6. and found that the tendency of the TA sound field distribution on the semicircular is invariable due to the increase of the emitting surface, but the amplitude of its TA sound pressure would increase, and the directivity of the TA wave is more accentuated, which was detected at *t* = 60 mm.

**Figure 7:**
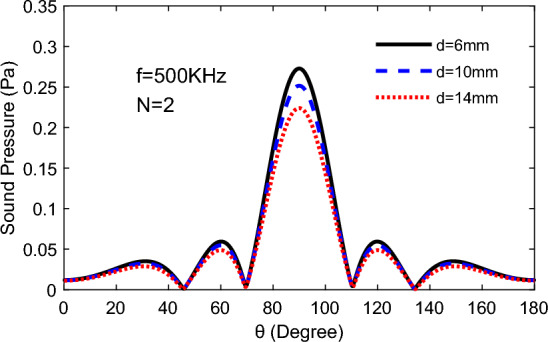
Comparison of TA sound pressure with altitude angle for different distance of emitting surfaces at the yoz plane: *t* = 60 mm.

According to the Fig. 7, the tendency of TA sound pressure on the semicircular remains consistent, but the sound pressure decreases as the distance of two emitter increase from 6 to 14 mm, owing to no change in the difference in distance from the measuring point to the two emitting surfaces.

While the above investigates the semicircular sound field distribution in the yoz plane perpendicular to the line pattern formed by the emitting plane, the following investigates the semicircular sound field distribution in the xoz plane.

**Figure 8:**
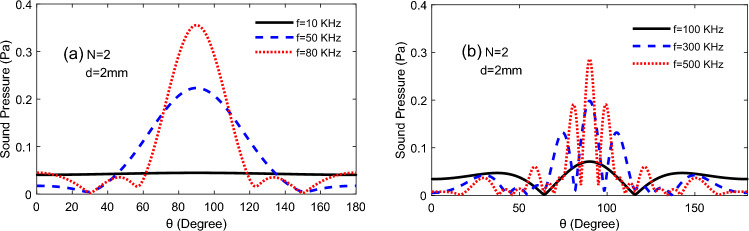
Comparison of TA sound pressure change with altitude angle at different input frequencies.

As depicted in Fig. 8. It was found that the distribution of the TA sound field on the semicircular became gradually complex as the heat frequency increased, and although the directivity was highlighted, the interference of the side lobes also became progressively frequent. Especially, the input frequency at *f* = 80 kHz, which is a reasonable frequency to enhance the directivity of the TA sound pressure.

#### The distribution of TA sound pressure based on the different phase

According to the model in Fig. 5b, we studied three emission surfaces, which array in a line and neglecting the distance of each other, that is *d* = 0, and *a* = 2 mm, *f* = 80 kHz, *t* = 60 mm. thus, The scanning of the TA sound field can be achieved from changing the difference of phase.

**Figure 9:**
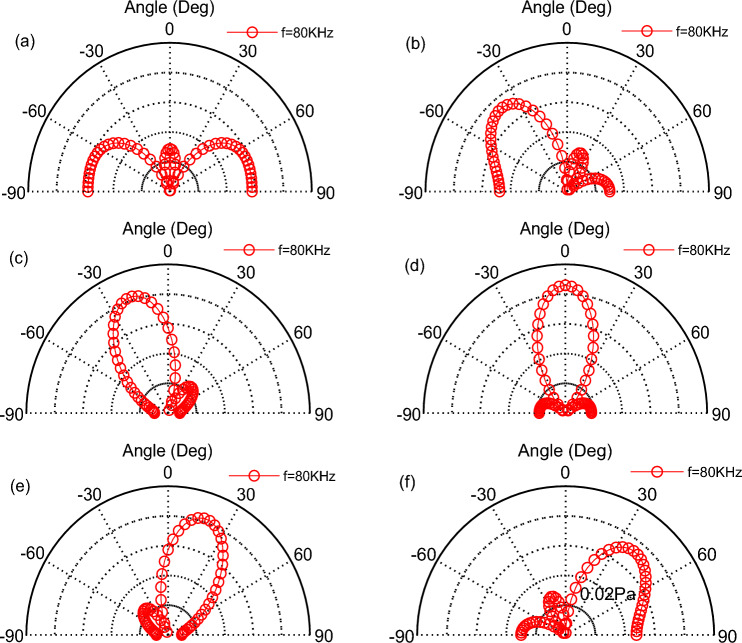
The scanning of TA sound waves in the 0°–180° direction at specific phases: (**a**) (− 180°,0°,180°); (**b**) (− 120°,0°,120°); (**c**) (− 60°,0°,60°); (**d**) (0°,0°,0°); (**e**) (60°,0°, − 60°); (**f**) (120°,0°, − 120°).

**Figure 10:**
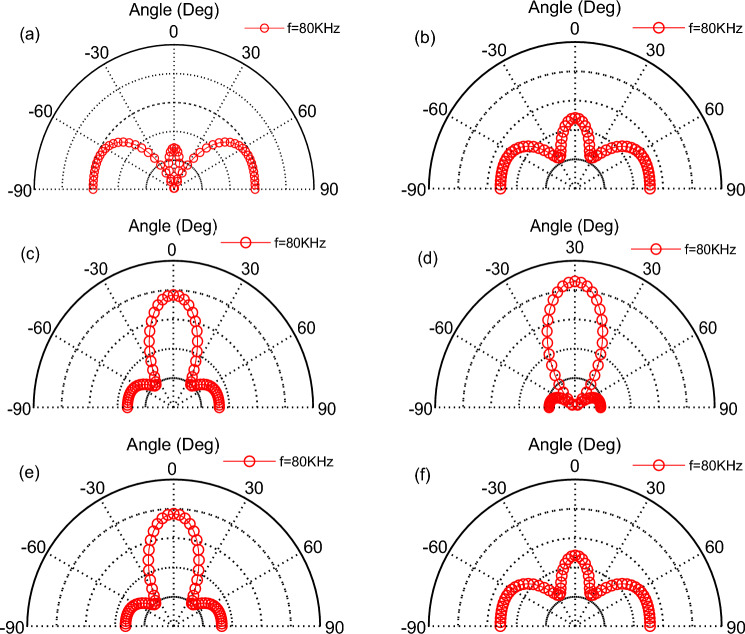
TA wave scanning in the 0°-180° direction with the Phase-symmetrically distribution.

The distribution of phase difference from (a)–(f) for each TA emission surface is in order: (− 180°,0°,180°) → (− 120°,0°,120°) → (− 60°,0°,60°) → (0°,0°,0°) → (60°,0°, − 60°) → (120°,0°, − 120°), which clearly displays the scanning of TA wave in the direction of 0°–180 in the Fig. 9. Then the phase of each TA emission in the above array is transformed, using a symmetric phase distribution: (180°,0°,180°) → (120°,0°,120°) → (60°,0°,60°) → (0°,0°,0°) → (60°,0°,60°) → (120°,0°,120°), which is shown in Fig. 10. The emission surfaces on both sides have the same initial phase, the direction of the phased array TA beam does not change. No scanning occurs with an input frequency of *f* = 80 kHz, and what changes is only the acoustic pressure of its TA wave. However, the TA emission surfaces under the symmetric phase distribution of the conditions do not form a TA wave scanning in the 0°–180° direction.

#### Effect of distance of emitting surfaces on TAPA

**Figure 11:**
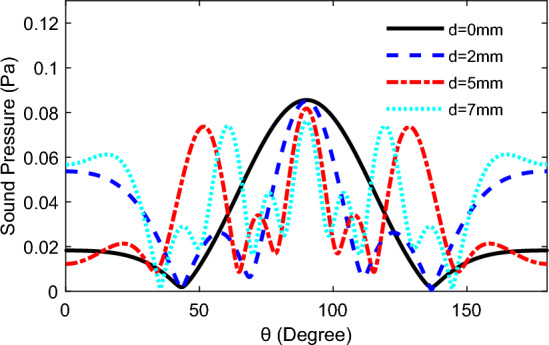
Variation of TA sound pressure with height angle *θ* for different TA surface distances.

The Fig. 11 shows the behaviour of TA sound pressure with altitude angle for three TA emitting surfaces arranged in a line pattern, with a distance *d* between the different emitting surfaces, and on a semi-circular circumference 60 mm from the centre of the array of TA emitting surfaces, which at an input frequency *f* = 80 kHz. From the figure, it can be seen that increasing the distance between the TA emission surfaces would enhance the fluctuation of the TA pressure and the increase the side lobes, furthermore, the tendency for the TA pressure at the altitude angle *θ* = 90° to decrease, and the magnitude of the TA pressure of the side lobes on both sides increases, which decreasing the directivity of the whole phased array.

**Figure 12:**
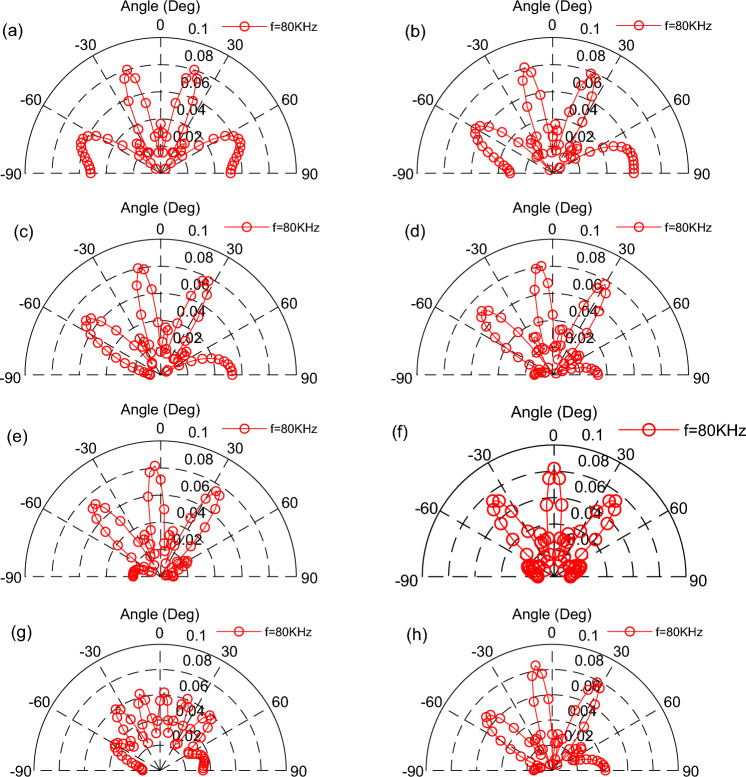
Scanning of TA waves in the 0°–180° direction at a distancing of 5 mm between TA emitting surfaces.

By changing the distance between the emitting surfaces while keeping the phase distribution of the three TA emitting surfaces unchanged as (− 144°,0°,144°) → (− 108°,0°,108°) → (− 72°,0°,72°) → (− 36°,0°,-36°) → (0°,0°,0°) → (0°,30°,60°) → (45°,90°,135°), the same input frequency, the distance between adjacent TA emission surfaces *d* increased to 5 mm. The phased array of TA waves in the 0°–180° direction of the scanning is shown in Fig. 12, which illustrates that the increase in the distance of the emitting surfaces would lead to the TA sound waves of the parabolic lobes increase, and narrowing the parabolic scanning angle, and it would be interact with each other.

More lobes appear in (h) for the asymmetrical phase angle, thus, increasing the distance between the TA emission surfaces is not conducive to the scanning of TA waves in the 0°-180° direction.

#### The area of emission surface affects the TA waves emitting

**Figure 13:**
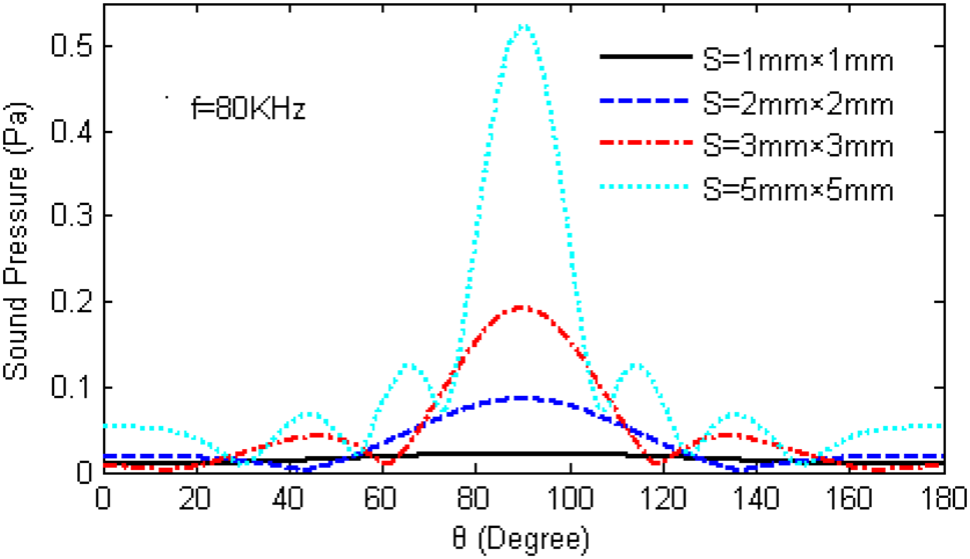
TA sound pressure changes with the altitude angle under different area of emitting surface.

As shown in Fig. 13, the directivity of TAPA increase clearly with the increased area, but would lead to the formation of multiple wave peaks of TA wave at the direction of 180°,and more peaks would appears as the increased area of the emitting surface.

**Figure 14:**
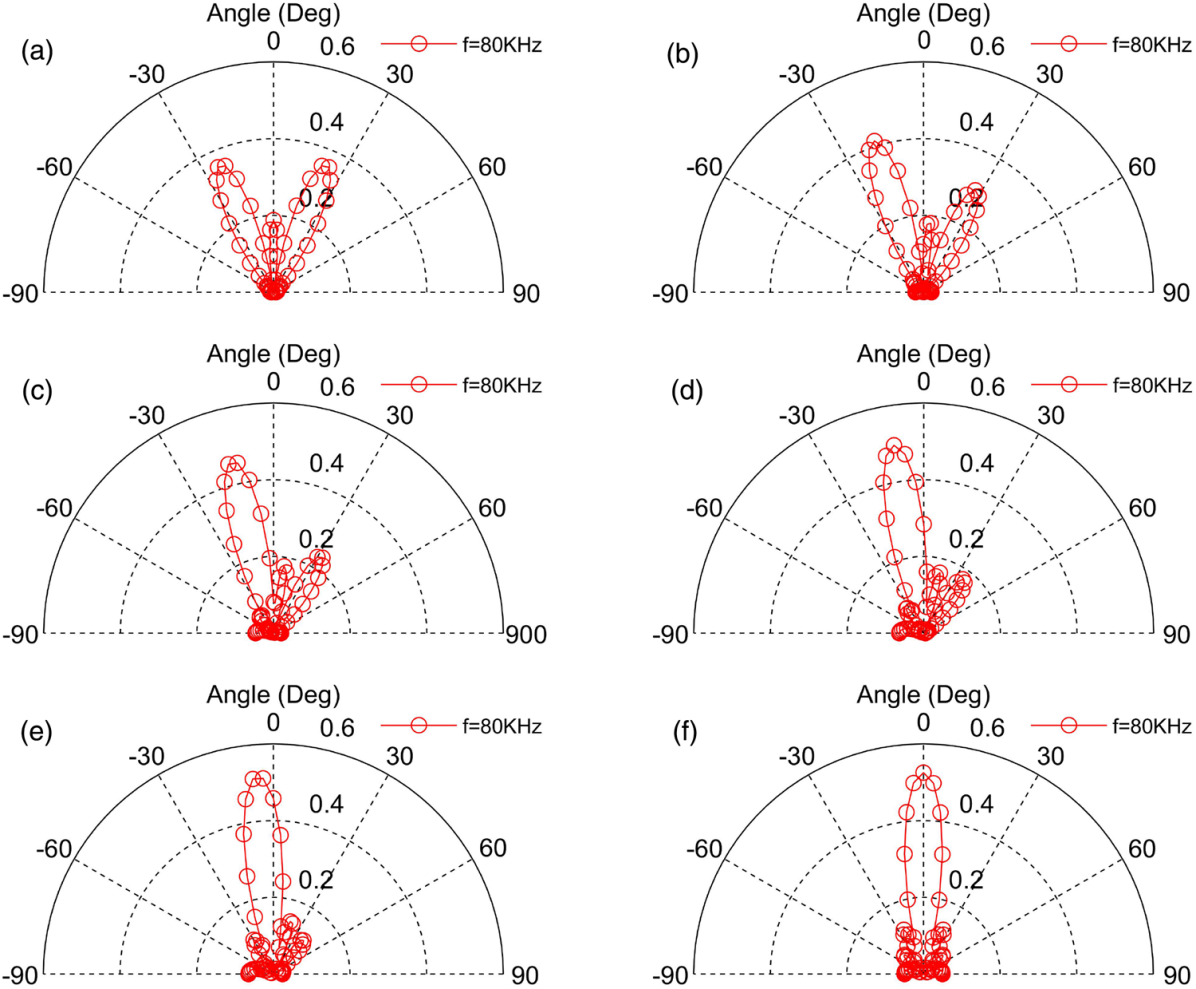
Scanning of TA waves in the 0°–180° direction by a phased array consisting of TA emission surfaces with an area of 25 mm^2^.

A line pattern TAPA consisting of three TA emitting surfaces with an area of 5 mm × 5 mm is shown in Fig. 14, which shown that increasing the area of the emitting surface while keeping the frequency and spacing of the TA emitting surfaces unchanged, the TA wave of the phased array is more directional and the energy is more concentrated, but the range of the TA wave scanning becomes narrower and there are multiple paraflaps.

#### The TA waves emitting varied by different quantity of emission surfaces

**Figure 15:**
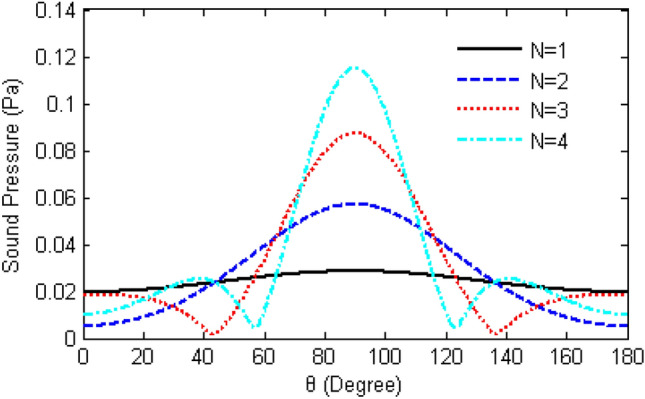
Variation of TA pressure with altitude angle for different numbers of TA emission surfaces.

The multiple TA emission surfaces are arranged closely in a line shape without spacing, with an area size of 2 mm × 2 mm, and under the condition of input frequency *f* = 80 kHz, by changing the number of TA emission surfaces, the TA pressure change on the half-circle circumference with the distance from the centre of the emission array surface of 60 mm is observed as shown in Fig. 15, and it can be seen from the figure that, with the increase in the number of TA emission surfaces, the entire TA phased array size also becomes larger, but the directivity of the emission array is getting better and better, then it will lead to an increase in the number of TA pressure peaks.

**Figure 16:**
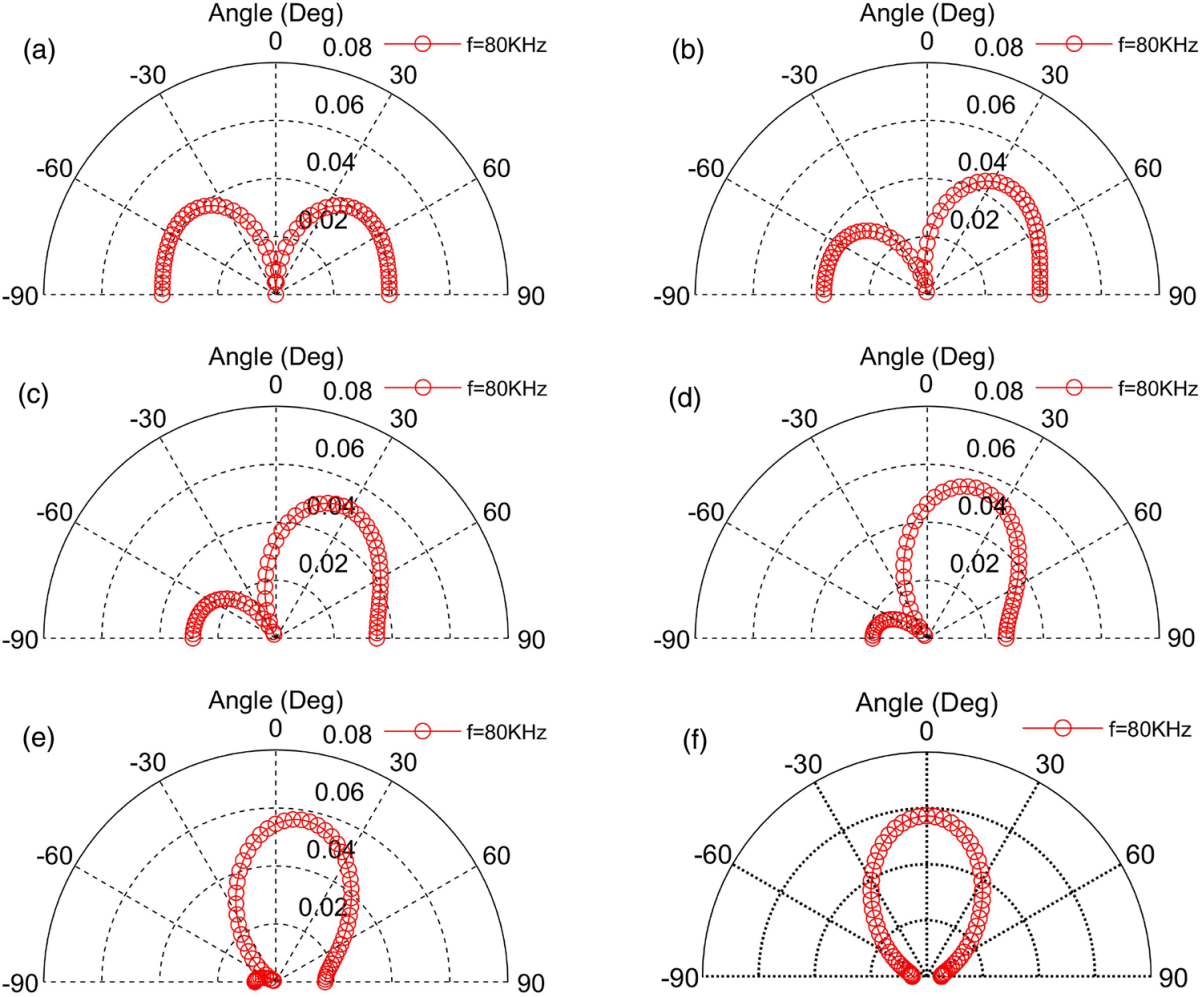
Scanning of a phased array of two TA emission surfaces in the 0°–180° direction.

**Figure 17:**
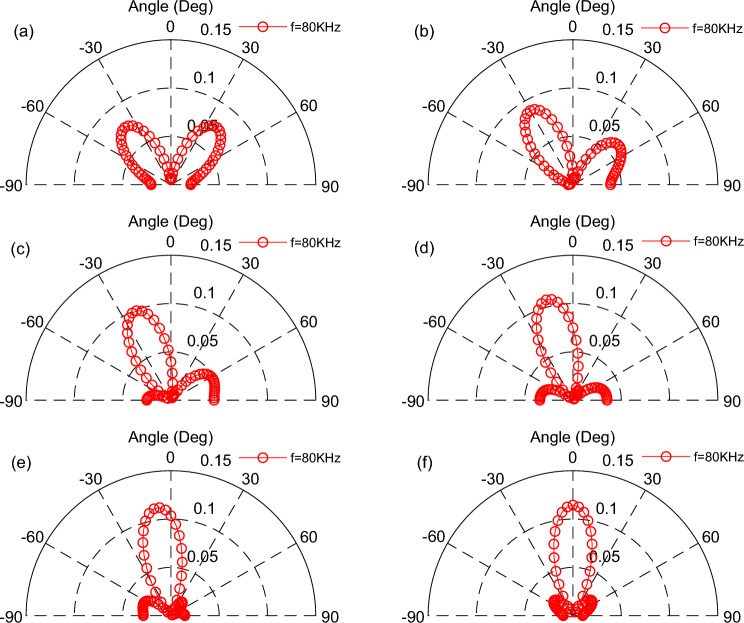
Scanning of a phased array of four TA emission surfaces in the 0°–180° direction.

The Figs. 16 and 17 represent a phased array of two and four square emitting surfaces with an area of 4 mm^2^ arranged in a shape of a line, respectively. For a TAPA composed of two TA emitting surfaces, the phase distributions of the emitting surfaces are as follows: (0°, − 180°) → (0°, − 144°) → (0°, − 108°) → (0°, − 72°) → (0°, − 36°) → (0°,0°). As for the TAPA composed of four TA emission surfaces, the phase distribution of each TA emission surface is as follows in order: (− 180°,0°, 0°,180°) → (− 144°,0°, 0°,144°) → (− 108°,0°, 0°,108°) → (− 72°,0°, 0°,72°) → (− 36°,0°, 0°,36°) → (0°,0°, 0°,0°), and the input heat flow frequency is *f* = 80 kHz. Comparing these two graphs and Fig. 9, it is easy to find that increasing the number of TA emitting surfaces would narrows the TA scanning range of the TAPA and the number of side lobes.

### The effect of arrangement of TA emission surfaces

**Figure 18:**
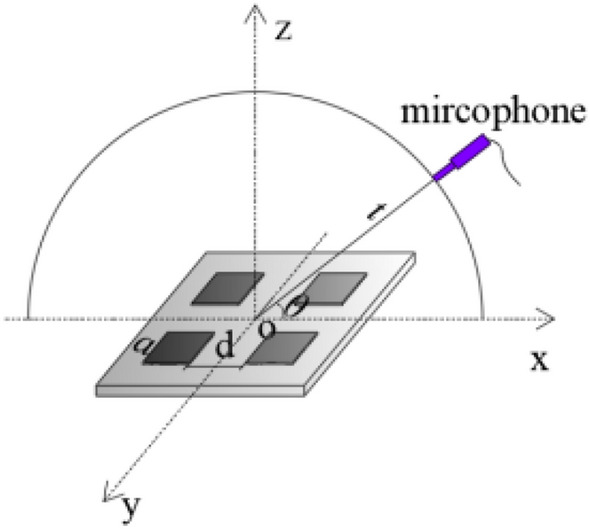
The basic schematic of four emitting surfaces arranged in a square array.

We have discussed the TA emission surfaces closely arranged in a line array and researched the multiple influencing factors of this arrangement clearly.Thus, we arranged four TA emission surfaces with a square array, as shown in Fig. 18, to study the effect of the distance between the elements and the size of the area on the TA emission.

**Figure 19:**
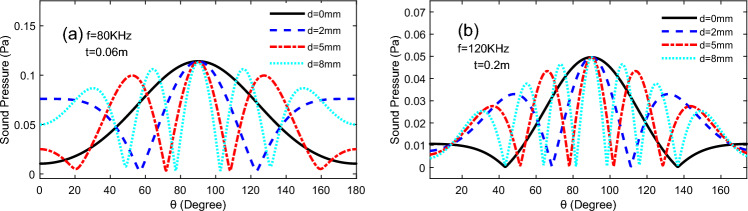
The variation of TA sound pressure with altitude angle for different distances of TA emission surfaces: (**a**) *f* = 80 kHz, *t* = 60 mm, a = 2 mm; (**b**) *f* = 120 kHz, *t* = 20 mm, a = 2 mm.

In Fig. 19, while keeping the area of the individual TA emission surfaces constant, as the distance between the TA emission surfaces increased, which increasing the side lobes of the TA waves produced by this TAPA, and enlarging the distance would affect the TA pressure of the side lobes, but the main flap magnitude of the TA pressure never varies with the increase in the distance between the emission surfaces.

**Figure 20:**
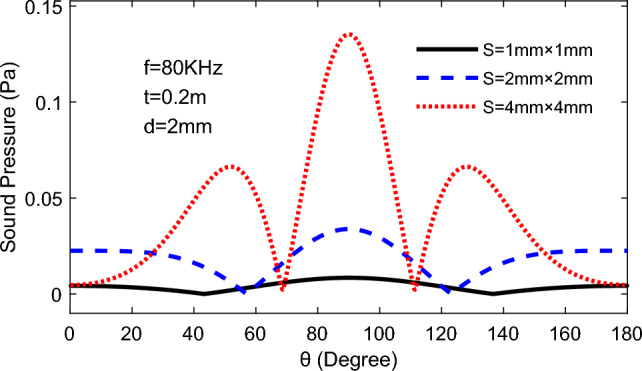
The variation of TA pressure with Altitude angle for different areas of unit TA emission surfaces: *f* = 80 kHz, *d* = 2 mm, *t* = 200 mm.

From the Fig. 20, it can be observed that when the square TA emission area of the unit is 1 mm^2^, there is only one wave peak on the curve of TA pressure variation with altitude angle, whereas when the area of a single square TA emission surface becomes 16 mm^2^, the curve contains three wave peaks. And increasing the area of emission surface will grow more side lobes, but the ability of TA emission of whole phased array is more concentrated, and the directivity will be more pronounced.

## Conclusion

Based on the previous theoretical study of TA emission, this chapter considers the influence of the sample and substrate. The method of point source integration is used to derive the acoustic pressure formula of the phased array TA emission, which serves as the theoretical basis for exploring the influences of different input heat flow frequencies, the quantity of TA emitting surfaces, the distance, the area, as well as the phase changes between the emitting surfaces and the arrangement of the acoustic wave distribution and scanning. The following conclusions were drawn.

1. The TA pressure distribution on the semi-circular circumference forms a standing ellipse, no side lobes when *a*/*λ* = 1, Increasing the frequency strengthen the directivity of the TA emission but also increase the quantity of side lobes.
2. A phased array consisting of two emitting surfaces shows the same trend of TA distribution in the semicircle between the two emitting surfaces as a single emitting surface. Increasing the distance between the emitting surfaces decreases the magnitude of the TA pressure but does not change the trend of the acoustic pressure distribution on that semicircle. The semicircular sound pressure distribution in the vertical square-section over the centres of the two emitting surfaces becomes increasingly complex with growing frequency, and the number of side lobes increases.
3. The TA phased arrays arranged in a line pattern have the applied phase distribution in the order of (− 180°,0°, 0°,180°) → (− 144°,0°, 0°,144°) → (− 108°,0°, 0°,108°) → (− 72°,0°, 0°,72°) → (− 36°,0°, 0°,36°) → (0°,0°, 0°,0°), which becomes narrower with the increase of the frequency of the input heat flow, the increase of the area of the TA emitting surface, and the increase of the number of emitting surfaces, and would enhance the directivity of TA wave and enlarge the number of side lobes. However, increasing the distance between the TA emitting surfaces will facilitate the growing of grating lobes, which is not beneficial to the scanning of the phased array, and reducing the TA pressure at the top of the centre of the array surface.
4. For a TA phased array arranged in a square shape, the change of the distance between the TA emission surfaces never affect the sound pressure magnitude of the main flap of the TA wave. Increasing the distance only lead growing in both the number and the size of the side lobes. The increase of the area of the TA emission surface will enhance the directivity of the TA wave and the influence of the side lobes. Thus in order to enhance the directivity of a square-shaped TAPA, it is necessary to reduce the distance of the emitting surfaces or increase the area of the emitting surfaces.

## Data Availability

The datasets used and/or analysed during the current study available from the corresponding author on reasonable request.
